# Exploring healthcare assistants’ role and experience in pain assessment and management for people with advanced dementia towards the end of life: a qualitative study

**DOI:** 10.1186/s12904-017-0184-1

**Published:** 2017-01-19

**Authors:** Bannin De Witt Jansen, Kevin Brazil, Peter Passmore, Hilary Buchanan, Doreen Maxwell, Sonja J. McIlfatrick, Sharon M. Morgan, Max Watson, Carole Parsons

**Affiliations:** 1 School of Pharmacy, Queen’s University Belfast, Belfast, UK; 2 School of Nursing and Midwifery, Belfast, UK; 3 Centre for Public Health, School of Medicine, Dentistry and Biomedical Sciences, Belfast, UK; 4 Patient and Public Involvement Representative, Carer for a person living with dementia, Belfast, UK; 5 Kerrsland Surgery, Belfast, UK; 6 Institute of Nursing and Health Research, Ulster University, Newtownabbey, UK; 7 All Ireland Institute of Hospice and Palliative Care, Our Lady’s Hospice and Care Services, Dublin, Ireland; 8 Marie Curie Hospice, Belfast, UK; 9 Northern Ireland Hospice, Belfast, UK; 10 School of Pharmacy, Queen’s University Belfast, 97 Lisburn Road, Belfast, BT9 7BL UK

**Keywords:** Dementia, Palliative care, Pain assessment, Pain management, Healthcare assistant

## Abstract

**Background:**

Pain assessment and management are key aspects in the care of people with dementia approaching the end of life but become challenging when patient self-report is impaired or unavailable. Best practice recommends the use of observational pain assessments for these patients; however, difficulties have been documented with health professionals’ use of these tools in the absence of additional collateral patient knowledge. No studies have explored the role, perspectives and experiences of healthcare assistants in pain assessment and management in dementia; this study provides insight into this important area.

**Methods:**

A qualitative approach was adopted, using key informant interviews with healthcare assistants caring for people with advanced dementia approaching the end of life in hospice, nursing home and acute care settings. Thematic analysis was the analytic approach taken to interpretation of interview data. Data were collected between June 2014 and September 2015.

**Results:**

Fourteen participants took part in the study. Participants’ average length of caring experience was 15.4 years and most were female. Three key themes emerged: recognising pain, reporting pain, and upskilling. Participants were often the first to notice obvious causes of pain and to detect changes in patient norms which signified hidden causes of pain. Comprehensive knowledge of resident norms enabled participants to observe for behavioural and nonverbal indicators of pain and distinguish these from non-pain related behaviours. Pain reporting was heavily impacted by relationships with professional staff and the extent to which participants felt valued in their role. Positive relationships resulted in comprehensive pain reports; negative relationships led to perfunctory or ambiguous reporting. Participants emphasised a desire for further training and upskilling, including in the use and reporting of basic pain tools.

**Conclusions:**

Healthcare assistants are frontline staff who have a key role in direct patient care, spending a considerable amount of time with patients in comparison to other health professionals. These staff are often first to notice changes in patients that may signify pain and to alert professional staff. However, to ensure the quality of these reports, further efforts must be made in reversing stigma attached to this role and in upskilling these members of the healthcare team.

## Background

Pain is prevalent among people with dementia, often resulting from advanced age and multiple comorbidities [1–4]. Pain recognition and assessment in this patient population is widely recognised to be challenging as extensive cognitive deterioration in advanced stages often significantly impairs or removes the possibility of patient self-report, exposing patients to the risks of under-assessment and under-treatment of pain [1, 5–8]. Where patient testimony is unavailable or extensively impaired, health professionals are advised to observe for behavioural and nonverbal cues (such as grimacing, guarding, frowning, moaning, agitation, and aggression) which may indicate pain in nonverbal, cognitively impaired adults [9].

Standardised pain assessment tools provide an organised format by which professionals can attribute an estimated pain severity score to each behavioural indicator and aggregate them on completion to provide an overall score of estimated pain in the patient [10, 11]. However, difficulties associated with the use of these tools have been reported due to the overlap of behavioural indicators with indicators of other, non-pain related conditions such as hunger, distress and boredom [12, 13]. It is therefore recommended that scores from these assessments be interpreted in the context of other collateral knowledge and information about patients [14, 15].

As nurses’ and physicians’ administrative workloads continue to increase and, as a consequence, time spent with patients is reduced, much of the direct care lies with healthcare assistants (HCAs) [16–18]. HCAs (also known as nurse auxiliaries, healthcare support workers, and personal/clinical support workers) work in health and social care settings providing care to patients across a range of physical and psychosocial domains usually under the supervision of Registered Nurses (RNs). Their typical duties include: providing personal care, maintaining patient hygiene, assisting patients with eating and toileting, providing social interaction and psychological support, and basic housekeeping (e.g. making beds, laying tables for meals) [16–21]. These staff spend more time with patients than any other healthcare professional and often develop detailed knowledge of patients’ preferences, routines, and normative patterns of behaviour, mood, appetite and disposition [19–22]. As a result, they are often the first to recognise and alert professional staff to changes in patients’ physical and cognitive functioning [22].

Previous work has explored the impact of HCAs on patient care and outcomes in the context of the care of older adults, palliative care and dementia [19–23]. No previous work has examined HCAs’ experiences and perspectives of and contributions to pain assessment and management in nonverbal patients with dementia approaching the end of life. This study aimed to explore HCAs’ perspectives and experiences of pain assessment and management in people with advanced dementia approaching the end of life in hospice, acute care and nursing home settings to address this gap in an area of clinical practice widely acknowledged to be challenging and critically important. It forms part of a wider programme of research into assessing and managing pain in this complex patient group, from which a number of articles have been published [24–27].

## Methods

### Study population and sample

Criterion purposive sampling with maximum variation (regarding age, educational attainment and care experience) was used to recruit HCAs who were caring for people with advanced dementia approaching the end of life or who had since died. In consideration of the range of contexts in which end of life care is provided for people with dementia, participants were recruited from hospice, secondary care and nursing home settings across Northern Ireland (NI), a province within the United Kingdom (UK). Index contacts comprising consultant physicians from acute care (geriatric medicine *n* = 2; palliative medicine *n* = 2 and psychiatry *n* = 1), Medical Directors of hospices (*n* = 4) and nursing home managers (*n* = 5), disseminated study information comprising a cover letter, participant information sheet and contact consent form via email or in hard copy to eligible HCAs. Subsequent settings suggested by these index contacts were followed up and study information disseminated as described above.

### Study design, data collection and analysis

Participants’ experiences and perspectives of pain assessment and management in advanced dementia were explored through semi-structured key informant interviews. This approach allowed participants flexibility to describe experiences and perspectives important to them within an overarching topic focus which enabled convergent and divergent themes to be identified and explored [28, 29]. The interview schedule comprised 10 questions developed through review of literature on pain assessment and management in dementia and studies of HCAs working in palliative care, dementia and other patient populations. Questions were reviewed and refined following consideration of discussion and feedback from members of the Project Management Group (PMG), thereby ensuring that the focus of the study was addressed. Examples of interview questions are presented in Table 1.

**Table 1:** Examples of interview questions and key themes supported by illustrative participant quotes

Example of interview questions	Key themes and participant quotes
How do you recognise/identify when a person with advanced dementia who is approaching their last few months, weeks, days and hours of life is in pain? Are there any factors that make it difficult to tell if someone with advanced dementia is in pain? How do you overcome these challenges?	***Theme: recognising pain*** *“We use a lot of body language to ascertain if they’re in pain. We had a gentleman recently who’d had a fall. He was refusing to eat and this went on for a few days and I actually phoned his GP. The gentleman was obviously in pain because he was in bed all day, every day, he was drawing his knees up, he was grimacing, rocking and certainly even just from the fact that he* *** must *** *have been* *** hungry *** *as well, we* *** knew *** *he had some form of pain”. HCA06 Nursing Home* *“It would be difficult if you didn’t know the patient, you know? Once the patient’s with you for a while you can read their moods and sometimes just by looking at them you know they’re in pain. It* *** would *** *be difficult if it was a new patient to you and you hadn’t got to see the wee signs or understand the non-verbals from the patient. It* *** would *** *be difficult then, yes”. HCA03 Hospice*
Describe your experiences of reporting and discussing pain in residents with advanced dementia with other healthcare professionals.	***Theme: reporting pain*** *“* ***Any *** *changes in a patient’s condition I refer to the nurse-in-charge”. HCA014 Hospital* *“I would go to a staff nurse or a doctor and say you: this seems different to me, this is unusual, do you think this patient would be in pain?” HCA03 Hospice* *“Whenever—see if a resident gets say paracetamol, well then you can be monitoring for like two to four hours afterward and if you feel that the person has settled, well then obviously it’s working but if the person hasn’t settled, well then obviously the paracetamol isn’t working on her. So we would go back then and again you contact the doctor”. HCA08 Nursing Home*
Do you think further/additional training in managing pain in patients with advanced dementia is required for healthcare professionals? How do you think this should be delivered?	***Theme: training and upskilling*** *“Maybe some ideas and tools that could be put in place to help us assess pain* *** appropriately *** *and deal with it appropriately”. HCA05 Hospice* *“I think we can be maybe that bit more* *** hands-on *** *with pain assessment when the nurse has, you know a lot to do with her role. We can be there by the bedside and as I say,* *** any *** *changes I would see in a patient’s condition, I would make sure I pass on”. HCA013 Hospital* *“We should, we should be given more training. Probably how to recognise it [pain] better cause’ there’s so many different ways to tell if somebody is in pain and if there was anything that* *** we *** *could instead of* *** always *** *having to go to the nurse”. HCA011 Nursing Home*

Data were collected between June 2014 and September 2015. Participants were interviewed at their place of work in rooms located away from other staff, patients and families. Prior to interview, participants were provided with a verbal summary of the aims and objectives of the project, the interview procedure, and procedures for the analysis, treatment and storage of the data, and any questions were addressed. All participants provided written informed consent to participate in the interviews and for the interviews to be digitally recorded. No monetary or other incentives were offered for participation in the study. Ethical approval for the study was obtained from the Office for Research Ethics Committees Northern Ireland (ORECNI); reference 14/NI/0013.

Verbatim transcripts of the digital recordings were made by the first author and independently verified against the recordings by two members of the PMG. All qualitative data were analysed using the thematic approach detailed by Braun and Clarke [30]. Using this approach, units of data were provisionally coded according to their content (experiences, thoughts, feelings etc.) and grouped by concept to form themes. These were subsequently reviewed against the raw data and assigned a descriptor to reflect their content. An audit trail of each step of analysis was kept and the method of data analysis and findings were discussed and agreed by members of the PMG in bi-monthly meetings [31].

## Results

Fourteen HCAs participated. Participants’ average age was 44.9 years (range: 20 to 62 years) and most were female (*n* = 13). Most participants had provided care for people with dementia in multiple settings over the course of their work life. Participants’ average length of caring experience was 15.4 years (range: 1 to 27 years). Participant characteristics are presented in Table 2. The key themes emerging from the data supported by participant quotes are presented in Table 1, and are further discussed below.

**Table 2:** Participant characteristics (*n*/%)

	*n* (%)
Gender	
Male	1 (7%)
Female	13 (93%)
Care setting (Specialty)	
Nursing Home	9 (64%
Acute care (hospital)	2 (14%)
Hospice	3 (31%)
Length of clinical experience	
Average	15.4 years
Range	1 year to 27 years

### Recognising pain

Most participants recognised pain to be a key care concern for people with dementia and emphasised a need for vigilance in identifying its presence, particularly for residents unable to self-report. Although not required to conduct formal pain assessments, HCAs’ narratives revealed that they regularly performed informal relationship-centred pain assessments using knowledge of residents acquired during the course of daily care provision. In so doing, HCAs identified behaviours, nonverbal cues and other activities which departed from residents’ daily norms, and interpreted these as potential indicators of pain, distress or health decline. A minority of participants; however, reported challenges recognising pain in this patient population. These themes are discussed in detail below.

#### Knowing the resident

Close daily contact with residents during the course of providing care resulted in participants accruing detailed comprehensive knowledge of their patients including their preferences, normative patterns of behaviours, moods, demeanour, cognitive and physical functioning and routines. Unusual or unexpected changes in these domains were interpreted as warnings of acute illness, pain, or other health decline, whilst changes in appetite and toileting routines prompted consideration of constipation and urinary tract infections. Care tasks which required moving, lifting, turning and providing personal care meant participants were often the first to notice obvious causes of pain such as contractures, bruises, cuts, lacerations and abrasions, pressure sores, rashes and other injuries.

It could be just that, you know, they’re maybe not using a *hand* the way they usually do, or as I say the way they *behave*, you know, it could be aggressive behaviour or it could be, as I say, going into their shell. Maybe they’ve stopped eating or they’re refusing to eat or they won’t go to the toilet or they’re going to the toilet *more*. It’s these things you just have to go into: is it because of infection? Or is it because of something else? *Knowing* the resident is the most important thing from A to Z as far as I’m concerned. (HCA01 Nursing Home)

When we were getting [the resident] up, she was really, really, really contracted [in] her arms. It was so bad you couldn’t get the hoist straps in…and I know from getting her up before on *other* days, it would have been easy. And it was something to do with her hands as well; she had wee sores [between] her fingers so we found them as well. So she was going through the pain of us holding her hand *and* lifting her up. (HCA012 Nursing Home)

#### Observing and interpreting behavioural and nonverbal indicators of pain

HCAs in this study were not required to conduct formal pain assessments and did not use standardised pain assessment tools; eleven participants reported being unfamiliar with their contents and application. However, participants’ narratives revealed that when pain was suspected in nonverbal patients and no obvious physical cause could be determined, most HCAs observed patients for many of the behavioural and nonverbal expressions of pain usually considered in such assessments. These observations were interpreted within the context of HCAs’ holistic knowledge of residents, which facilitated distinction between expressions of pain and other, non-pain related states (e.g. boredom, hunger). Participants also reported that pain recognition was facilitated in residents who exhibited characteristic expressions of pain which only manifested at intermittent intervals and disappeared following administration of pain relief. Most participants believed that learning to recognise and interpret the ways in which residents expressed pain was critical but reported difficulty in doing so for newly admitted residents with whom they were unfamiliar. In these cases, HCAs sought additional information regarding patients’ normative behaviours and/or behaviours, activities or nonverbal cues known to be expressions of pain from residents’ referral letters, nursing notes and anecdotal information provided by nurses and residents’ families, friends and social workers. Three HCAs regularly accompanied nurses during administration of the Abbey Pain Scale; these narratives revealed disparities between HCAs’ and nurses’ knowledge of residents and participants emphasised the importance of interpreting scores within the context of patient norms. A minority (*n* = 4) of participants, however, expressed task-oriented attitudes to care and reported feeling under-educated in recognising behavioural and nonverbal indicators of pain. These HCAs used nurses’ reports of pain to adapt care routines to accommodate pre-existing pain rather than proactively monitoring for new occurrences of pain, and did not regularly liaise with residents’ families.

Most of the time they’re [residents] unable to *tell* us so therefore we’re looking for non-verbal facial expressions, movement of the hands, grimacing of the face and also times where they’re nearly putting their body into the foetal position. That would alert us to pain. Um, *agitation*, if they’re agitated then we try to work out if it could be pain causing that. We also would look to *families* because they’ve been looking after them and they may recognise that it’s when the [resident’s] left hand comes up to their head that we will know that they’re in pain. (HCA04 Hospice)

A lot of the time some of them can be *between* scores - do you know what I mean? And it does depend on the person who’s doing it, if the nurse is doing it and they don’t really know [the resident] as well, the nurse could say: well she does usually move this much and I would say: no, she *doesn't* usually move that much. So that’s why when they’re doing the Abbey Pain Scale, one of us would be with [the nurse] as well. (HCA08 Nursing Home)

Interviewer: How would you recognise pain in someone with advanced dementia approaching the end of life?

HCA011: You know, just facial, noise, sometimes movement. I would say there’s [sic] probably other ways but I don’t know. (HCA011 Nursing Home)

### Reporting pain

All HCAs reported pain to nursing staff within their setting. However, the quality of pain reporting varied according to HCAs’ approaches and attitudes to care, their relationships with other health professionals and the extent to which they felt recognised and valued within the team. HCAs’ narratives revealed positive and negative relationships with other healthcare professionals and their perspectives on inclusion in, and exclusion from, multidisciplinary team (MDT) meetings.

#### Positive work-related identities and relationships

Positive work-related identities and relationships with other healthcare staff were formed when HCAs felt encouraged and supported in raising and discussing their concerns about patients (including but not limited to pain), and where they felt qualified professionals recognised and valued their role. These staff provided detailed reports which described their observations and offered a rationale for suspecting pain. They believed that quality in pain reporting resulted in their concerns being taken seriously and allowed patients to be assessed quickly. These participants were consequently motivated to monitor for and report on patients’ responses to analgesia which they believed was necessary to alert nursing staff to cases of potentially unresolved pain. They adapted care routines, postponing tasks that involved touching, lifting, moving or turning residents until after analgesia had taken effect.

We had this gentleman who was coming to the end of life. I said to the nurse, he’s got to be sore; he’s making all these noises, facial expressions as well, the frowning and the hand up (gestures stop signal with her hand), trying to stop you touching [him]. Straight away the doctor was called, the prescription was written and that was it. Life was comfortable for him after that. (HCA02 Nursing Home)

If a pain relief *hasn't* been effective, we will then go and say to the nurse we don’t think that has worked and then they will follow that up with the GP. (HCA010 Nursing Home)

I would leave whatever we’re doing, if we’re getting her [the resident] up in the morning, I would leave her for 15 min or until the painkillers actually kick in before I go back and start getting her up and everything like that. (HCA012 Hospice)

#### Negative work-related identities and relationships

Five participants employed in nursing homes reported problematic relationships with nursing staff and/or physicians. This occurred when HCAs perceived other health professionals to emphasise and maintain professional hierarchies which created distance between these professionals and HCAs, leaving the latter feeling ignored and under- or even de-valued and resulting in communication breakdown among staff. Negative relationships with other health professionals resulted in pain reporting that was perfunctory, ambiguous or uninformative. HCAs who reported these relationships did not engage in monitoring or reporting treatment response or adapting care routines to take account of onset of analgesic effect. No participants were invited to attend or participate in MDT meetings; this was a cause of disappointment and frustration even among those who reported positive work relationships. Experienced HCAs believed that they missed out on critical information exchange that could inform patient care, and that their omission, as frontline staff, weakened the entire healthcare team. Others expressed frustration at being unable to contribute to care discussions despite their significant knowledge and understanding of residents. These participants expressed a belief that the lack of professionalism attached to their role (i.e. being unqualified, unregistered) was a key factor in being omitted from MDT meetings.

Sometimes doctors don’t like you telling them what you think, you know, because I’m only a care assistant, you know? (HCA01 Nursing Home)

I’d go and tell them ‘uns [ones] and just say I don’t think he’s well and then they [nursing staff] come down and assess him and whatnot. (HCA09 Nursing Home).

Sometimes it can be a wee bit frustrating for us as healthcare assistants because we have *so much* contact with the patients, we would have a *lot* more really than the qualified nurses would have. I mean their focus is mainly on administering medications and there’s other duties that they have that we’re not carrying out, but *we're* there with the patients a *lot* more and we know them quite well and we get to *know* when their pain is inclined to be particularly bad or worsen or be better. (HCA04 Hospice)

#### Upskilling

Thirteen participants believed that HCAs required ongoing, formal, needs-driven training in order to manage the demands of their role and provide high standards of care. One participant believed that most necessary knowledge and skills were developed through performance of the role, negating the need for additional training. The majority felt strongly that provision of excellent standards of care was dependent on the knowledge, skills and ability of all healthcare staff, including those providing care at the frontline. Many felt they could make a greater contribution to pain assessment and management in advanced dementia and much interest was expressed in learning to use and report basic assessments such as the Abbey Pain Scale, and in receiving formal instruction on how to monitor for and report treatment response, side and adverse effects. HCAs believed that the use of standardised tools would improve consistency in HCA pain reporting to health professionals. None of the participants anticipated that their use of assessment tools would replace or take precedence over assessments conducted by qualified staff. E-learning, the most common platform for delivery of training to HCAs in this study, was widely and heavily criticised for delivering generic, unengaging content of limited utility which failed to address their learning needs.

Any extra training is beneficial for our patients as well ourselves, you know, even if *we* were to do the Abbey Scale, you know, or something like that. (HCA012 Hospice)

If we had a trained nurse to teach us what to look for, like treatment response, and if they could show us the signs and explain to us how to interpret [them], that would beneficial. (HCA06 Nursing Home)

I just find that the e-learning especially, it’s just very boring and really time-consuming and then I do get bored of it and I’m like [sighs loudly] I don’t want wanna ever read through that, so I skip that, whereas face-to-face training is more interesting, they can make it fun and it’s not just reading off the computer screen. (HCA07 Nursing Home)

## Discussion

The complexity and challenges of pain assessment and management for people with advanced dementia are well-recognised and documented with much of this previous work focused on the experiences of nurses and physicians on whom the responsibility of these assessments usually falls [12, 32, 33]. To our knowledge, this is the first study which explores HCAs’ perspectives and experiences of, and their role in, pain assessment and management for people with advanced dementia approaching the end of life.

HCAs in this study were not required to conduct formal pain assessments for their residents and most reported being unfamiliar with the contents and application of standardised pain assessment tools. However, participants’ narratives revealed that most regularly performed informal relationship-centred pain assessments as an inherent part of care provision. Such assessments occur when knowledge and understanding of patients’ normative patterns of behaviour, physical and cognitive functioning and past reactions to pain are used to inform recognition and interpretation of behavioural and nonverbal pain cues [34]. Daily care provision brought HCAs in this study into close physical and social contact with residents, allowing them to develop a comprehensive knowledge and understanding of their care-recipients over time [19–22]. In most cases, changes in residents’ norms prompted HCAs to observe for behavioural and nonverbal indictors of pain and interpret them within contextual knowledge of the resident. Closer analysis revealed that most were performing many of the observations required by standardised assessment tools recommended by healthcare policies for use in this patient population [10, 11, 35–38]. Participants’ knowledge of residents also allowed idiosyncratic expressions of pain to be recognised as such and facilitated distinction of behavioural indicators of pain from non-pain related cues, overcoming a commonly reported difficulty associated with pain assessment in nonverbal patient populations [12, 32]. Understanding the residents for whom they cared was perceived to be critical to recognising and reporting pain, illness or distress in these patients, particularly for those unable to self-report. Many participants in this study reported difficulties and limitations in recognising pain in residents with whom they were unfamiliar. Previous research reported similar findings for nurses and certified nursing assistants (CNAs) [34]. Interestingly, in the present study, most HCAs sought to gain insight into residents’ past pain experiences and behaviours through liaison with patients’ families, friends and other key health and social care staff. The involvement of families and other key social contacts in pain assessment for patients with severe cognitive impairment and/or dementia for whom self-report is unavailable, has been widely recommended [39–43].

Findings in this study illustrated three cases of disparities between HCAs’ and nurses’ knowledge of residents which may have critical implications for the interpretation of pain scores. The finding reported here suggests that both staff should be present during assessment to allow outcomes to be considered alongside other collateral patient information [14, 15, 44]. Participants with task-oriented approaches to care were a minority in this study. These staff reported being under-skilled in recognising pain and relied largely on nurses’ reports of existing pain to approach care routines with additional caution, rather than attempting to observe for signs of new pain. Previous studies have reported poor patient and staff outcomes which result when healthcare staff lack understanding of the pain experience in people with dementia and are inadequately trained and supported to recognise, assess and manage pain for these patients [45–48].

Pain reporting was heavily impacted by the nature of relationships with other healthcare staff. Participants who were openly encouraged and supported to raise and discuss all concerns, including pain, and who felt valued for their contribution to patient care provided detailed reports which expounded their interpretations of observed behaviours [49]. These HCAs monitored for and reported back on patient response to analgesia and, without nurse direction, planned care tasks to allow the benefits of pain relief to manifest before attempting to move, lift or turn patients. Positive work-related identities and relationships with other staff have resulted in improved staff morale, increased confidence and improvements to care quality and patient outcomes [19–21, 49]. Negative relationships were reported in cases where professional hierarchies dominated and participants felt ignored or undervalued. Strong in-group identities among HCAs can propagate ‘us and them’ attitudes leading to negative perceptions of and noticeable professional distance from nursing and medical staff; a finding reported in another study of HCAs in working in dementia care [21]. Pain reporting was severely impacted by negative work-related identities and relationships; in such cases it was at best perfunctory and detail-poor, and at worst uninformative, with participants referring to general ill health rather than pain. Work-related identity construction is recognised to impact significantly on patient care and outcomes and has been studied extensively using social identity theory [50–56]. Negative work-related identities, strong in-group membership and dysfunctional team dynamics are associated with deterioration of collaborative approaches to care and withholding or poor exchange of information, exacerbating the challenges of complex care and resulting in negative outcomes for patients and staff [21, 57–60]. The impact of the quality of communication between healthcare professionals on patient outcomes has been studied extensively across multiple health conditions including dementia; much of this previous work has focused on nurse-physician interactions [60–62]. Although this study provides insight into HCA reporting of pain, the findings are limited to their perspectives alone. Future studies may wish to examine nurses’ and physicians’ experiences of and perspectives on communicating with HCAs, or explore communication dyads and triads among these staff in order to elucidate how information provided by HCAs is received, understood and processed by qualified health professionals. This is particularly important given that tensions in relationships between CNAs and RNs have been previously reported [34].

Participants expressed frustration and disappointment at being excluded from MDT meetings with many believing this resulted from stigma regarding their status as unqualified workers. Most believed the benefits of HCA participation in the MDT would be reciprocal with all members of the team benefitting from having access to patient information that could inform care practice. Lloyd and colleagues (2011) warn that exclusion may result in HCAs feeling isolated from the wider care team, strengthening in-group identities and lead to difficulties as described above [21]. The importance of inclusivity and teamwork among health professionals is emphasised in a number of health policies and is a required competency of the HCA role [63–68]. Future research could evaluate the impact of the inclusion of these staff in the multidisciplinary team on patient care and team dynamics, and should include the perspectives and experiences of HCAs as well as other members of the MDT.

The large majority of participants believed that ongoing, needs-driven training which actively enhances knowledge and practical skills is required for all professionals and frontline staff involved in the care of people with dementia. Interestingly, the minority of participants who provided sparse answers for other interview questions responded comprehensively when asked whether further training was required. Appropriately educating staff to understand the complexities of dementia care results in better management of the emotional and physical demands of the work, increased engagement with the role and adoption of person-centred approaches to care [69]. Healthcare policy emphasises a need for all staff (including HCAs) working in dementia to be appropriately educated, trained and equipped to competently provide high quality care but does not suggest appropriate platforms for delivery of this training [70, 71]. Most participants reported that e-learning was unengaging, generic and resulted in little, or no, skills and knowledge development. These findings are reiterations of those in many other studies indicating little progress in this area and suggesting the need for a continued effort towards developing and trialling engaging and appropriate educational programmes for these staff [22, 23, 72]. Few studies have been completed in this regard; however, positive outcomes for HCAs’ confidence, motivation to engage in care and reporting to professionals have been reported following practical skills training in palliative care [73]. Many participants believed that the HCA role could be expanded in dementia care; a sentiment echoed in recent work [74]. In the current study, most participants (including task-oriented HCAs) expressed significant interest in learning how to monitor for and report on treatment response, side and adverse effects, and how to use and report basic assessments such as the Abbey Pain Scale to standardise and improve the quality of reporting among HCAs. Tools such as the Abbey Pain Scale are simple to use and do not require extensive training; previous work reports no significant difference in the ability of qualified health professionals and unqualified staff working outside the healthcare setting to detect pain in facial expressions [75]. However, given the challenges experienced by nursing and other health professionals in the use of these tools, and that assessment scores are often interpreted in conjunction with clinical judgement and collateral information from multiple sources, a number of factors must be considered when exploring an expanded role for HCAs in pain assessment. Firstly there is the question of how training should be delivered, and by whom. Secondly, given the variation in knowledge, skills and competence of HCAs, the process of selecting staff for this expanded role, the selection criteria used and how competence is determined must be considered. The manner in which this might be implemented in clinical practice without duplicating or complicating current use of pain tools and approaches to pain assessment, and without creating and/or exacerbating interdisciplinary tensions, requires careful consideration. Finally, there must be formal, robust evaluation of the use of pain tools by HCAs and the impact on patient care and outcomes, the culture of multidisciplinary working and approaches to end of life care across healthcare settings.

There are some limitations to this study. Although we aimed to recruit for maximum variation among participants, recruitment relied on contacting participants through networks established by members of the PMG. Acute and hospice care settings in this study are linked to teaching hospitals and a university; therefore it is likely that staff are better aware of pain in people with dementia than those in settings without connections to academic teaching and research. The majority of HCAs were recruited from nursing home settings; the shortfall of HCAs from acute care reflects the difficulties in interviewing staff working in this setting, whilst limited numbers recruited from hospice resulted from low take up of participation among these staff despite attempts to publicise the study in these organisations. Although data analysis did not identify significant difference between perspectives and experiences across care settings for most findings, negative relationships were only reported in the nursing home setting. Future studies may wish to expand on this study using a broader selection of HCAs across settings. The self-selecting nature of the sample means that the views of engaged, motivated participants with an aptitude for providing person-centred care may be overrepresented. However, whilst findings related to specific elements of pain assessment and management may reflect more instances of best practice than general practice among HCAs, many of the general principles regarding the way these elements are impacted by social and group identities, dynamics and relationships are supported by other studies of staff working in palliative and dementia care and other patient populations.

## Conclusions

Assessing and managing pain in dementia is a priority in provision of end of life care for people with dementia but is one of the most commonly reported challenges among nursing and medical staff. HCAs provide the majority of direct care to these patients, attending to a broad spectrum of physical and emotional needs, and as a result are often well placed to recognise problems as they arise. However, this study indicates that whether this critical information is shared with professional staff and the quality of that information exchange are highly dependent on individual and group identities which form in response to relationships with professional staff. This study highlights the potential for the large workforce of non-qualified healthcare staff to contribute meaningfully to a critical and complex area of dementia care. However, with the prevalence of people living and dying with dementia ever increasing, it is essential that serious consideration be given to, and progress made in, supporting, educating, training and upskilling these staff to improve the quality of care and care experience for people with dementia and their families. Critical to this is changing perceptions and the stigma that the HCA role is unqualified, poorly remunerated manual labour, reconceptualising it as a role which requires significant dedication, commitment and the resilience to respond to and meet the physical and emotional demands of caring for people with dementia with compassion and respect.

## Abbreviations

CNA: Certified Nursing Assistant
HCA: Healthcare assistant
MDT: Multidisciplinary Team
NI: Northern Ireland
ORECNI: Office for Research Ethics Committees Northern Ireland
PMG: Project Management Group
RN: Registered Nurse
UK: United Kingdom
